# Design and tribological performance of short S-Glass fibre reinforced biocomposites on the influence of fibre length and concentration

**DOI:** 10.1038/s41598-023-28645-6

**Published:** 2023-01-25

**Authors:** Raju, Chee Wah Loy, Kiho Cho, Paul Farrar, B. Gangadhara Prusty

**Affiliations:** 1 School of Mechanical and Manufacturing Engineering, UNSW Sydney, Sydney, NSW 2052 Australia; 2 Dental Materials Science, Division of Applied Oral Sciences and Community Dental Care, Faculty of Dentistry, The University of Hong Kong, Hong Kong SAR, China; 3 SDI Limited, Bayswater, VIC 3153 Australia; 4 ARC Training Centre for Automated Manufacture of Advanced Composites, UNSW Sydney, Sydney, NSW 2052 Australia

**Keywords:** Mechanical engineering, Biomedical materials

## Abstract

Fibre-reinforced biocomposites usage has gained prominence over the past decade. Although higher fracture toughness was observed when fibres were added to biocomposites, material degradation could occur due to filler and fibre content intolerance in the biocomposite matrix. Optimisation of resin-fibre-filler ratios helps in increasing the tribological performance of high load-bearing applications. However, the tribological performance is less understood due to limited in-vitro studies on the effect of fibre microstructures. A comprehensive investigation of the reciprocating and rotary wear behaviour of different compositions was carried out by varying fibre (0%, 5%, 10% and 15%) to particulate filler (40%, 45%, 50%, and 55%) weight fractions. The investigation aimed to identify the optimal composition of fibre-reinforced biocomposites based on the in-vitro ball-on-disc reciprocating and rotary wear tests in the presence of modified Fusayama solution. The cross-sectional areas of wear tracks were analysed using laser microscopy and scanning electron microscopy techniques to assess the surface morphology and subsurface damage of the wear tracks on biocomposites and the antagonist. The numerical results were statistically analysed using two-way ANOVA followed by a posthoc Tukey’s test (p = 0.05). The results showed a combination of adhesive, abrasive and fatigue wear for all the tested Groups. The friction coefficient had a longer transient period for the 5 wt% and 10 wt% Groups. Based on the surface roughness, coefficient of friction, SEMs, specific wear rate, and ease of manufacturing, the threshold limit for fibre loading was found to be 10 wt%. The rotary test had a considerably lower specific wear rate compared to the reciprocating test. Fibre weight fraction was found to be the influencing factor of the abrasive wear behaviour compared to fibre length for the tested Groups.

## Introduction

Advanced polymeric materials with new processing technologies play a key role to substitute the orthopaedic and dental parts of the human body. Biocompatible resin-based polymers are potential alternatives to currently used ceramic- and metallic-based prostheses that are in direct skeletal contact. The main reason for the high demand for polymer-based materials in orthopaedic implants is because of their low elastic modulus which is close to the human cortical (7–25 GPa) and cancellous (0.05–0.5 GPa) bones^1^. Various biocompatible polymer systems used in the manufacture of biocomposites comprise polyhydroxyalkanoates, polylactic acid, polycaprolactone, polyetheretherketone, polycaprolactone, polyurethane, polyethylene, methacrylates, polysulfone, and biopolymers such as gelatin, chitosan and alginate^2,3^. However, their low inherent mechanical properties, especially fracture strength in their pure form, limit their performance in biomedical applications. To overcome this problem, several inorganic reinforcements, such as metal, glass and ceramic particles and fibres are added to manufacture polymer composites which also enhance stiffness and toughness^4,5^. Also, the size and shape of these inorganic fillers directly influence the properties of the composite which include nano, micro and macro-sized particles. Nano-sized particles contribute to enhanced wear resistance but suffer from low fracture toughness^6^. Prior studies showed that commercial polymer materials reinforced with small quantities of randomly oriented short glass fibres along with particulate fillers significantly improved their strength and fracture toughness in comparison with conventional particulates reinforced polymer materials^7,8^. However, the physical and mechanical properties of the fibres, such as diameter, material, length, aspect ratio (AR = length to diameter ratio), tensile strength, orientation, weight fraction (wt%) and adhesion of reinforcing fibre strongly impact the physio-mechanical properties of light-curable polymer composites^9,10^. For example, the small quantity of randomly oriented short fibres along with micro/nano/hybrid fillers facilitate quasi-isotropic material. Although increasing the weight fraction of fibres can enhance the flexural strength and fracture toughness of polymer composites, other properties such as flowability and surface roughness could be compromised^11^. Furthermore, understanding the wear behaviour of artificial implant materials is one of the primary factors that need to be considered for load-bearing applications. While the addition of fibres in the biocomposites may enhance mechanical properties such as fracture toughness, wear resistance could be adversely affected, and optimisation of resin-filler-fibre parameters is required. The shape and size of the inorganic fillers have a significant influence on the load-bearing capability of the structure along with the tribological performance. It was also shown that the addition of glass fibres induced abrasive wear as the broken fibres act as abrasive particles and increased the wear rate^12^.

The polymerisation of these biocomposite resins could either be done chemically (two-part resin and hardener mixture) or via light-cured (UV, visible light) or thermal curing. Mixtures of urethane dimethacrylate (UDMA)/triethylene-glycol dimethacrylate (TEGDMA) monomers have shown a superior rate of polymerisation at a greater degree of conversion^13,14^. The main reason for better polymerisation is because of their low monomeric glass transition temperatures, for instance, TEGDMA (− 81.7 °C) and UDMA (− 41.7 °C)^15^. Among these compositions, light-curing 80 wt% UDMA and 20 wt% TEGDMA-monomer mixture has been considered as one of the suitable candidates for biomedical applications and some of the mechanical and physical parameters of the exact composition have been evaluated^16–18^.

Though in-vitro studies do not completely replicate in-vivo behaviour, considerable similarities in wear performance are observed when the factors influencing wear are isolated and repeatability is ensured^19^. Various parameters influence the measured wear from in-vitro tests such as resin composition, extent of polymerisation (degree of conversion), polymerisation shrinkage, antagonist material, sliding velocity, lubricating medium, applied load, duration of test and number of test cycles^20,21^. Filler size, filler volume and fibre dimensions are known to have a significant impact on the wear performance of resin-based biocomposites; the smaller the filler size with high volume, the higher the wear resistance^22,23^.

Many studies^24–28^ have focussed on the wear performance of reinforced biocomposites with either particulate composites (high wear resistance and low fracture toughness) or short fibres (low wear resistance and high fracture toughness). However, no studies have focused on substituting particulate fillers with short fibres without changing the total weight fraction of particulate fillers and short fibres in the overall biocomposite composition. Attention needs to be paid to the effect of the weight ratio of particulate fillers and short fibres on the properties of reinforced biocomposites, as this is one of the crucial parameters affecting the durability, cost, mechanical and handling properties of reinforced biocomposites. Optimisation of polymer-particulate filler-short fibre composition would provide better wear properties to the biocomposites in the load-bearing application. The aims of this study are to understand the effect of partial replacement of fibres with fillers on the wear performance of the biocomposite and to correlate the fibre-to-filler weight fraction and fibre aspect ratio with the wear resistance of the particulate polymer composites.

## Materials and methods

S-Glass fibres (S-2 Glass fibres) of 5 µm diameter from AGY, cut to lengths 250, 350, and 500 μm (Engineered Fibres Technology, LLC, USA) were used in this study. S-2 Glass fibre has a tensile strength of 4.89 GPa, Young’s modulus of 86.9 GPa, density of 2.46 g/cm^3^ and a refractive index of 1.52 at room temperature^29^. Based on parametric studies on the glass fibre etching and silane treatment from our earlier studies, *We *^18,30,31^ concluded that the technique with glass fibres etched in 37% HCl solution for 4 h and grafting with a silane coupling agent (3-(trimethoxysilyl)propyl methacrylate) for 1 h provided best results for flexural properties as well as ensuring random orientation of the fibres, by analysing the cross-section of the fractured surface. The same approach of fibre etching, silane treatment and mixing process is used in this study.

For the experimental composites, various weight fractions of silane-treated strontium aluminoborosilicate (SrO-Al_2_O_3_-B_2_O_3_-SiO_2_) glass fillers (0.7 µm) were dispersed in a monomer blend (80 wt% UDMA + 20 wt% TEGDMA). A photoinitiator (0.2% Camphorquinone), co-initiator (0.5% ethyl 4-dimethylaminobenzoate), tertiary amine system (0.05% 2,6-di-*t*-butyl-4-methyl phenol) and 1.6% fumed silica (Aerosil® R202, Evonik Industries) were added to monomer blend. Three different fibre lengths of 250, 350 and 500 µm with corresponding AR of 50, 70 and 100 respectively were etched in 37% HCl for 4 h and grafted with a silane coupling agent (3-(trimethoxysilyl)propyl methacrylate) for 1 h. Uniform mixing of each paste was done by FlackTek Speedmixer (Model DAC150FV, FlackTek Inc.) at 3500 rpm twice for 20 s under vacuum (to remove air bubbles). Ten Groups of experimental compositions (Group A to J) were prepared as shown in Table 1. Resin weight fraction and combined particulate filler and glass fibre weight fraction were kept at 45% and 55% respectively for all Groups. Samples with 5 wt% and 10 wt% fibres had better manufacturing ease in comparison with 15 wt% fibres which were a thick paste and small fibre lumps were visible with multiple void formations. Based on our earlier studies ^16,17^, the material properties range of the tested groups are: Flexure strength 120–143 MPa, Flexure modulus 5.5–6.5 GPa, Compression strength 280–330 MPa, Microhardness (VHN) of ~ 35, and thermal expansion coefficient 58–63 (*10^–6^)/deg C.

**Table 1:** Experimental test matrix.

Group	Type of wear test	Weight percentage of resin (wt%)	Weight percentage of filler (wt%)	Ø5 µm fibre		
				Weight percentage (wt%)	Length (µm)	AR
A-No Fibre	Reciprocating	45	55	0	NA	NA
B-5% 50AR	Reciprocating	45	50	5	250	50
C-5% 70AR	Reciprocating	45	50	5	350	70
D-5% 100AR	Reciprocating	45	50	5	500	100
E-10% 50AR	Reciprocating	45	45	10	250	50
F-10% 70AR	Reciprocating	45	45	10	350	70
G-10% 100AR	Reciprocating	45	45	10	500	100
H-15% 50AR	Reciprocating	45	40	15	250	50
I-15% 70AR	Reciprocating	45	40	15	350	70
J-15% 100AR	Reciprocating	45	40	15	500	100
K-No Fibre	Rotary	45	55	0	NA	NA
L-5% 70AR	Rotary	45	50	5	350	70
M-10% 70AR	Rotary	45	45	10	350	70
N-15% 70AR	Rotary	45	40	15	350	70

Standard wear samples 18 mm (L) × 10 mm (W) × 2 mm (t) were manufactured in a split stainless-steel mould where the prepared biocomposite paste was added and was made flush with the mould surface with glass slides on both sides. The samples were photocured using a curing light (Radii Plus, SDI) having an intensity of 1500 mW/cm^2^ for 60 s on each side. To minimise the effect of resin-rich behaviour on the sliding surface, all samples were subjected to grinding with 2000 and 4000 grit SiC abrasive papers and subsequently polished with 3.0, 1.0 and 0.04 µm diamond paste on a grinding/polishing machine (Struers Labopol-5). Polished samples were immersed in a sonicator bath with distilled water (Unisonics Australia FXP8D) for 6 min at room temperature to remove any grinding/polishing debris, dried with compressed air for 60 s and then stored in distilled water at 37 °C for a minimum of 24 h before the tests.

Two-body in-vitro reciprocating and rotary (unidirectional) wear tests (n = 5) were performed on a UMT-2 tribometer (CETR/Bruker) at room temperature (~ 20 °C) with modified Fusayama solution as the lubricating medium. Modified Fusayama’s solution^32^ was used to prepare the modified Fusayama solution to mimic the bio fluids. Reciprocating wear tests were conducted for Group A to J samples with a sliding distance of 30 m, a normal load of 20 N, a sliding velocity of 0.01 m/s, and a stroke of 5 mm at a frequency of 1.0 Hz. Rotary wear tests were conducted for Groups K to N samples with a sliding distance of 30 m, a normal load of 20 N, a wear track diameter of 6.4 mm and an angular velocity of 60 rpm. A 4.0 mm diameter 316-stainless steel ball (precision grade 200) was used as the antagonist. Stainless steel is one of the widely accepted biocompatible materials and is also affordable to the common public. Same material (biocomposite) antagonist would have provided limited insight into the tribological parameters and hence steel was used. The antagonist and modified Fusayama solution were replaced after each test and stored for further analysis.

The instantaneous coefficient of friction (CoF) and wear track depth data were collected from the UMT-2 tribometer software. The experimental datasets of CoF, wear-track width and depth and specific wear rate were statistically analysed with a two-way analysis of variance (ANOVA) (p = 0.05) followed by a posthoc Tukey’s test to find the relationship between the Groups.

Following the wear tests, the samples were dried at room temperature for a minimum of 24 h. The wear track cross-sectional area and surface roughness were measured using a laser microscope (KEYENCE, model VK-X200 series) with an objective lens of 20 × magnification at a resolution of 0.05 µm. The scanned images were processed using the KEYENCE VK-9700 software. The wear-track cross-sectional area and wear-track width for each Group of samples were evaluated by averaging the wear-track cross-sectional area and wear-track width obtained from the cross-sectional profiles at three to four different points on the wear track. Specific wear rate *S* (mm^3^/Nm) was calculated using the formula $S=\frac{W}{PVT}$^33^, where *W* is the wear track volume (average wear track area X wear-track length) in mm^3^, *P* is the applied load in N, *V* is the sliding velocity in m/s and *T* is the duration of the test in s.

A Hitachi TM4000Plus benchtop SEM was used to perform scanning electron microscopy (SEM) imaging. Before the SEM test, samples were sputter-coated with 10 nm platinum to prevent charging of the samples. SEM images were analysed qualitatively to obtain the surface and cross-sectional morphologies of the wear tracks. The steel antagonist surface was also analysed through SEM. To study the wear-track subsurface damage, the samples were cut, turned 90 deg, polished, platinum-coated and observed under SEM.

## Results

### Wear track width, depth and coefficient of friction (CoF)

**Figure 1:**
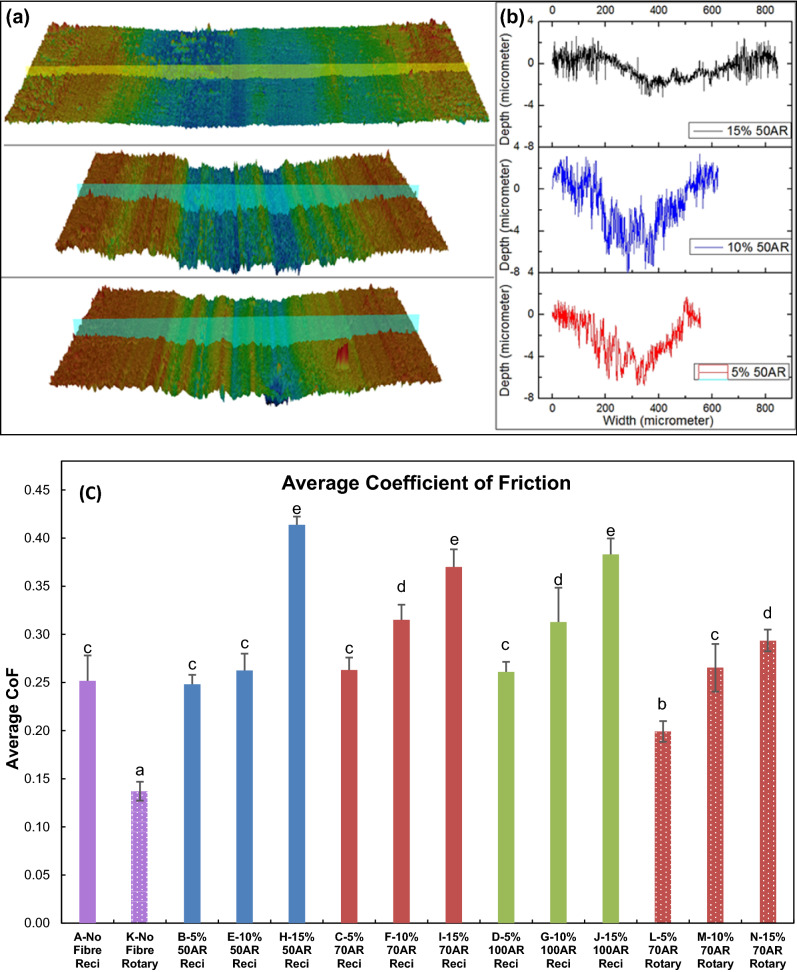
(**a**) 3D topography and (**b**) 2D profile across typical wear track of 50AR groups B (5 wt%), E (10 wt%) and H (15 wt%). (**c**) Average CoF for the reciprocating (Group A-J) and rotary tests (Group K-N).

A representative three-dimensional and two-dimensional surface topography wear tracks for 50 AR Groups are presented in Fig. 1a,b, respectively. As is seen from the topographical scanned images of the wear tracks demonstrate the intensity of wear along with qualitative and quantitative analysis of wear track width and depth. The wear scar depth was significantly lower for the 15 wt% Groups compared to the 5 wt% and 10 wt% Groups, while the 10 wt% Groups had the deepest wear scar.

For the reciprocating tests, the average CoF was observed to be the lowest for Group A (0.24) and in the range of 0.25–0.32 for Groups 5 wt% and 10 wt% fibre loadings as shown in Fig. 1c. All the Groups with 15 wt% fibre loading had a higher average CoF up to 0.41. Two-way ANOVA showed that the population means of fibre weight were significantly different (p < 0.01), however, it was not significantly different for fibre AR (p > 0.05). The interaction between fibre weight and fibre AR was significant (p < 0.01). Tukey’s post hoc test showed that the mean difference was not different between 5 and 10 wt%, but significant between 5 and 15 wt% and 10 and 15 wt%. For all the three fibre ARs within the same fibre loading Groups, the means difference was not significant (p > 0.05) showing the limited effect of fibre length on CoF. For the rotary test Groups, the average CoF were lower in comparison with the reciprocating test Groups. For No-filler composition, the rotary test average CoF was 0.14, which is lower compared to the reciprocating test average CoF of 0.24. This trend was observed in all the rotary test Groups with 70 AR. One-way ANOVA for rotary tests showed that the population means of fibre weight were significantly different (p < 0.01).

**Figure 2:**
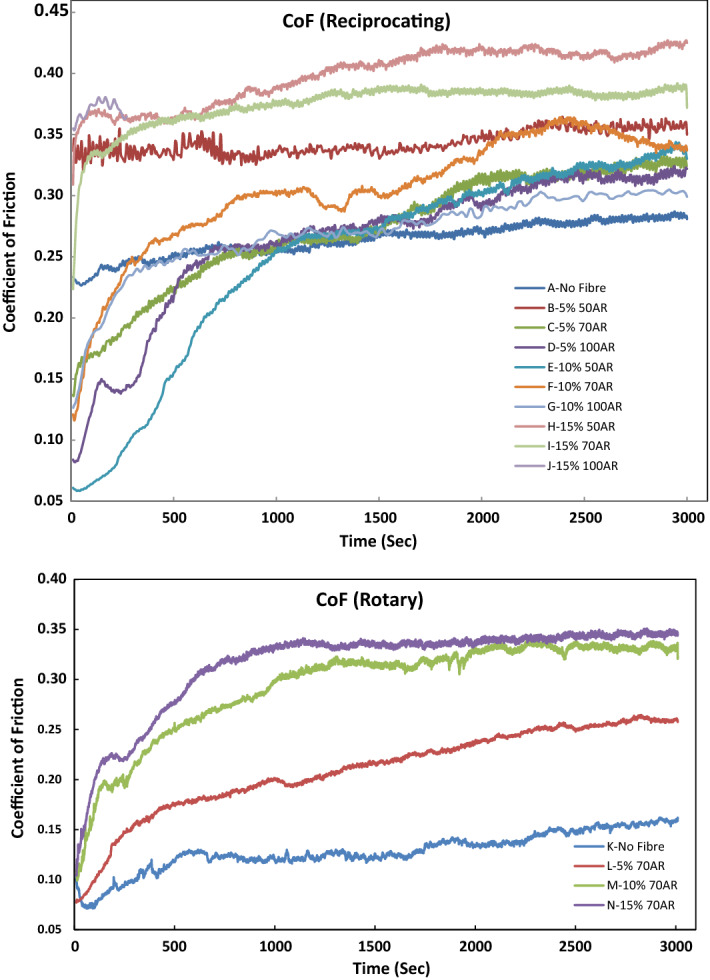
Transient and steady-state coefficient of friction vs. time for (**a**) reciprocating and (**b**) rotary tests.

Representative curves revealing the evolution of CoF with time for all the tested Groups are presented in Fig. 2. For the reciprocating test in Fig. 2a,b, Group A was seen to have a very short transient period (< 100 s) and a steady and linear increase of CoF while Groups B-E had a longer transient period up to 750 s. Groups B-E were also seen to have a higher rate of increase in CoF. However, Groups F-J had a shorter transient period up to 230 s and lower rates of increase in CoF. For the rotary tests, Group K with no fibre followed a similar trend of reciprocating test with a short transient period of CoF. Group L had a steady increase in CoF while Groups M and N seem to have stabilised CoF after the initial longer transient period of 1300 s. However, the average CoF for the rotary test was seen to be lower than the reciprocating test Groups.

### Specific wear rate

**Figure 3:**
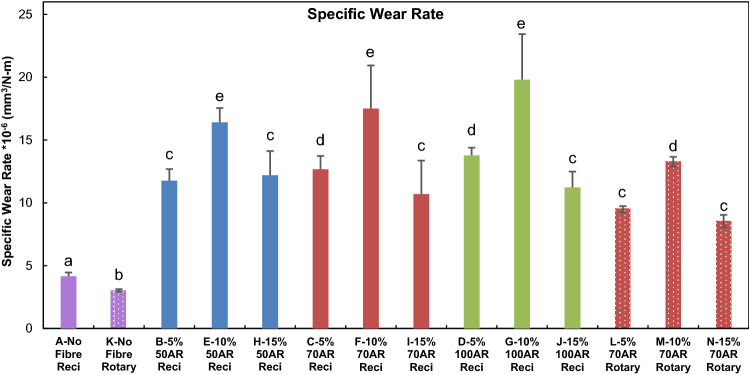
The specific wear rate of tested Groups along with the corresponding standard deviation. Groups with the same alphabets are not significantly different from each other (p > 0.05).

The calculated mean specific wear rate and standard deviation of the tested Groups are presented in Fig. 3. Groups shown with similar alphabets correspond to results that are not significantly different from each other. Two-way ANOVA (p = 0.05) followed by posthoc Tukey’s test revealed that the means difference was not significant between 50 AR-100 AR and 70 AR-100 AR, and the means difference was significant between 50 AR–100 AR, 5–10 wt%, 5–15 wt% and 10–15 wt%. Also, at the p = 0.05 level, the population means of fibre AR were significantly different, the population means of fibre weight fraction were significantly different and the interaction between fibre AR and weight fraction were also significantly different. Control Group A, in the absence of reinforcing fibres, had the minimum specific wear rate of 4.16 × 10^–6^ mm^3^/Nm. The specific wear rate increased from 5 to 10 wt% fibre loading but decreased from 10 to 15 wt% fibres. For 5 wt% loading, 100 AR had the highest mean wear rate followed by 70 AR and 50 AR. For 10 wt% loading, the mean wear rate was maximum for 70 AR and 100 AR. However, for 15 wt% loadings, the mean wear rate was almost the same for all three fibre lengths. The mean wear rate for all Groups under the rotary test was lower than the reciprocating test, with a lower standard deviation. For rotary test Groups, statistical analysis revealed that the means difference was significant for Groups K, L and N (p < 0.01).

### Wear track surface morphology using SEM

**Figure 4:**
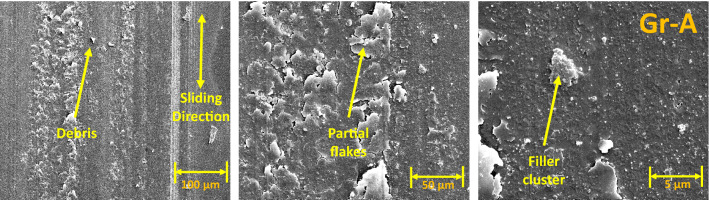
SEM images of Group A wear tracks.

**Figure 5:**
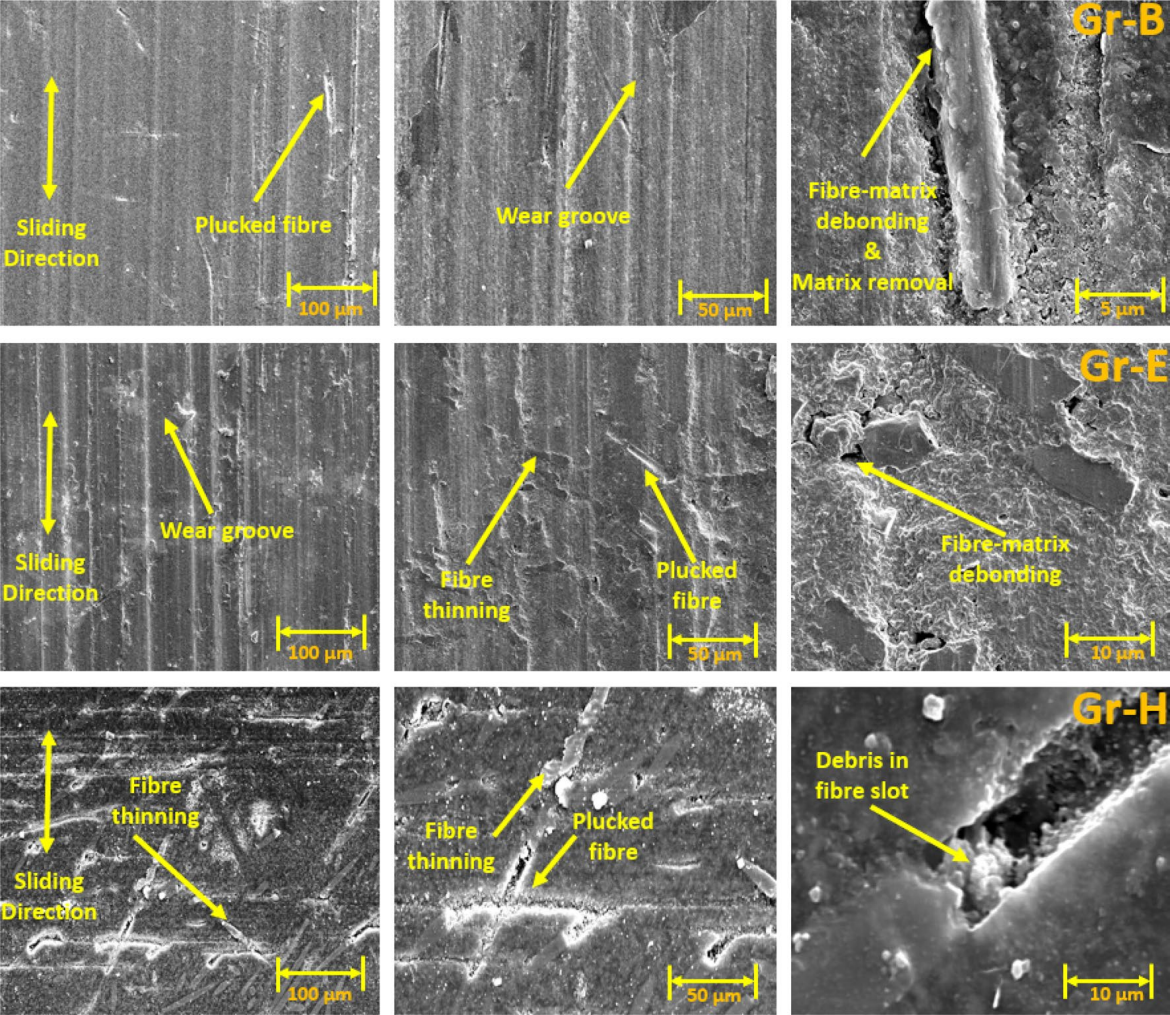
SEM images of 50 AR Group B (5 wt%), Group E (10 wt%) and Group H (15 wt%).

**Figure 6:**
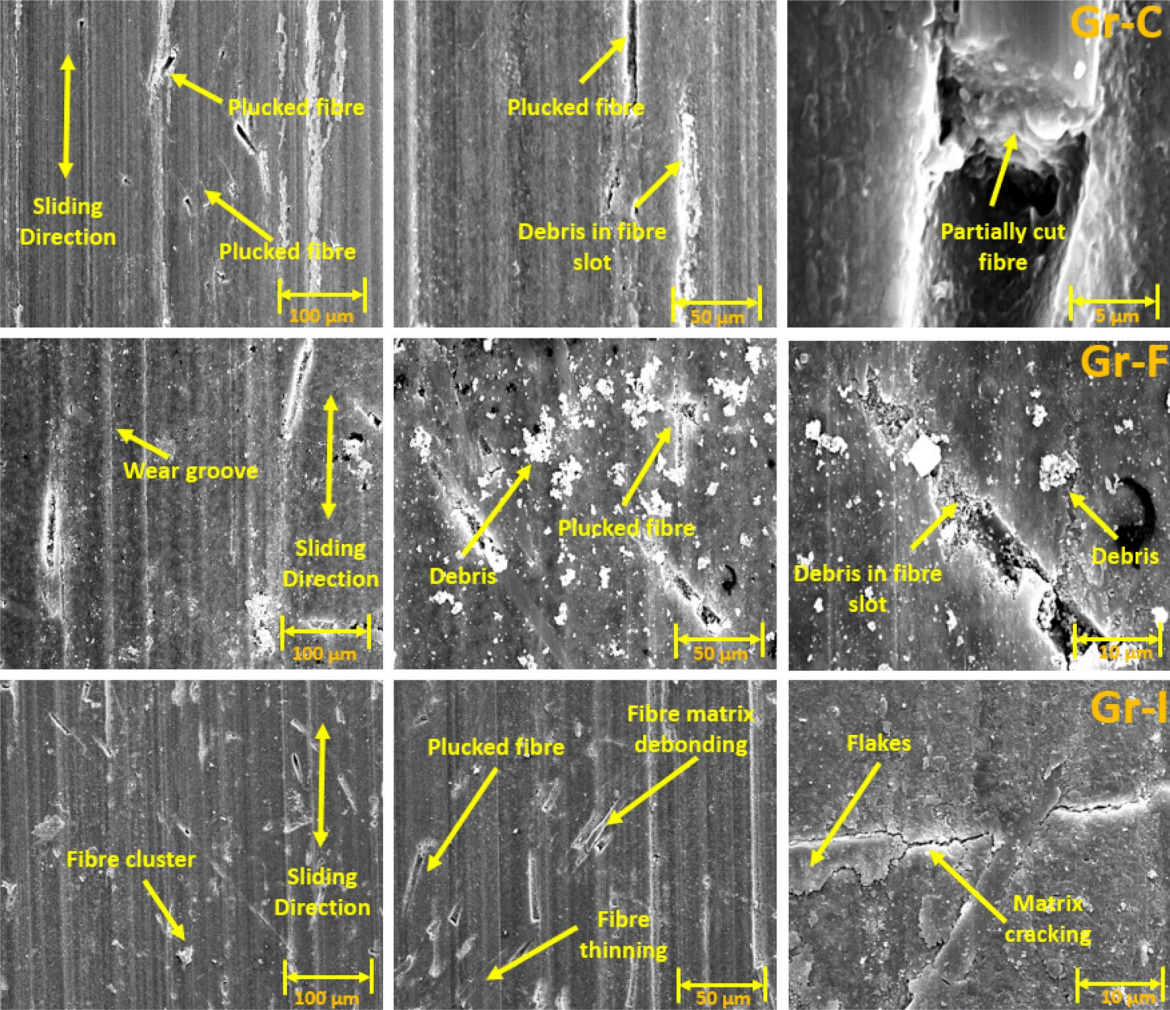
SEM images of 70 AR Group C (5 wt%), Group F (10 wt%) and the Group I (15 wt%).

**Figure 7:**
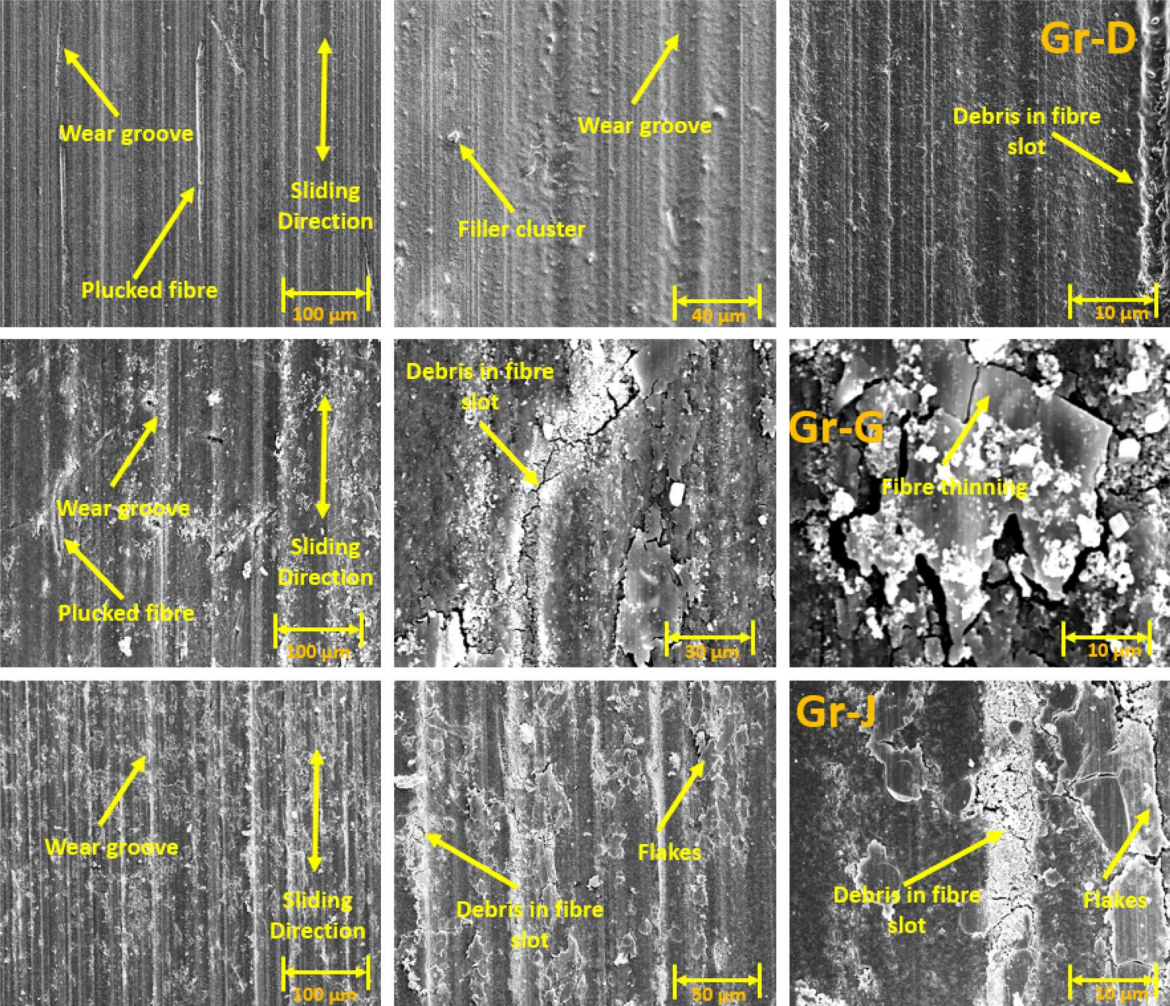
SEM images of 100 AR Group D (5 wt%), Group G (10 wt%) and Group J (15 wt%).

The SEM images of wear tracks for the reciprocating wear-tested Groups are shown in Figs. 4, 5, 6 and 7. Wear debris (polymer and filler) were observed on the wear tracks of all Groups (Group A-J). Rough surfaces with multiple debris, flakes and filler clusters are seen on the wear tracks of Group A in Fig. 4 which represents the fatigue wear mechanism. For the fibre-reinforced Groups, cavities created by the broken fibres were partially filled with debris. Partially embedded fibres were noticed on the wear track while any fractured fibres are likely to be washed away by the modified Fusayama solution. Matrix cracking around the fibre and removal of matrix around the fibre due to wear is also seen. All the 5 wt% and 10 wt% Groups have wear grooves along the wear track which shows an abrasive wear mechanism while the 15 wt% Groups had reduced wear grooves and increased number of flakes on the wear tracks showing combined fatigue and abrasive wear mechanism.

**Figure 8:**
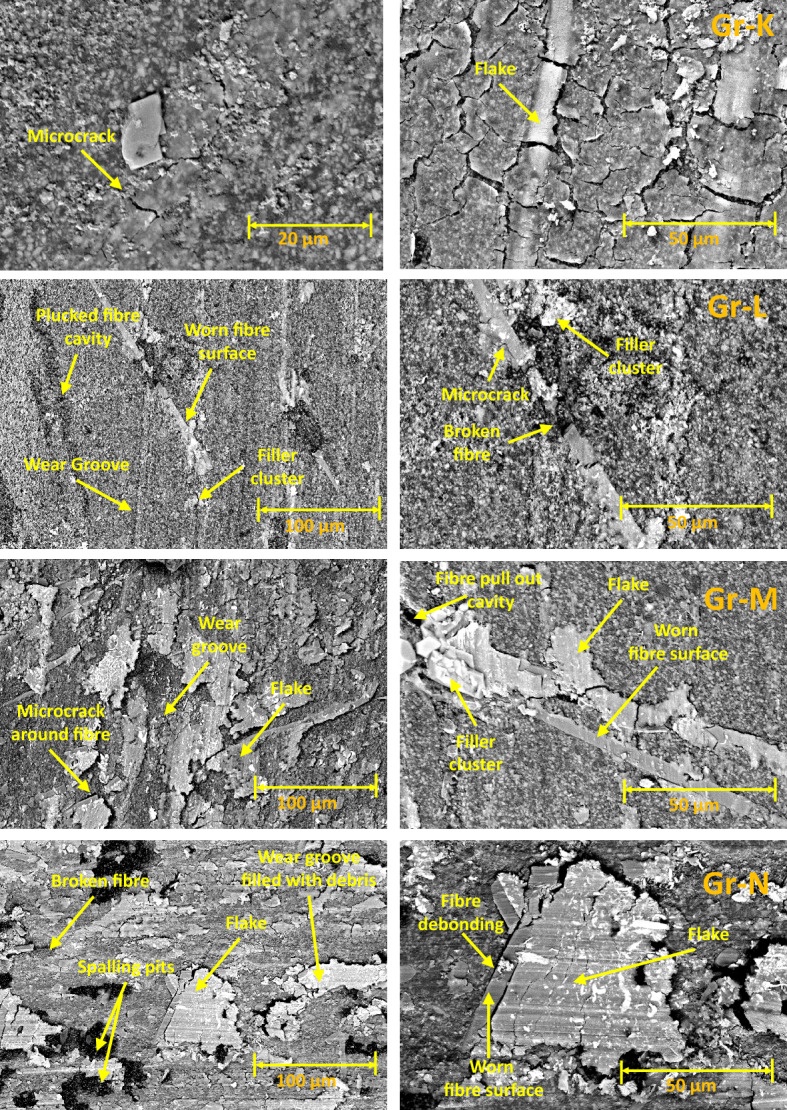
SEM images of wear surface for Rotary Test (Groups K-N): No Fibre, 5% 50AR, 5% 70AR and 5% 100 AR samples.

The SEM images of wear tracks for the rotary wear-tested Group are given in Fig. 8. Wear flakes, grooves, and debris were observed on the wear tracks of all Groups (Group K-N). Similar to the reciprocating test, the SEM images for Group K show a significant amount of wear flakes, subsurface cracks, pile-up, and sink-in features on the wear track. These features are related to the high degree of plastic deformation because of surface fatigue^34^ similar to reciprocating tests. The SEM images for Group L show a lesser number of flakes compared to Group K. This is due to the presence of 5 wt% fibre interlocking the resin matrix, improving the fatigue resistance of the biocomposite, and inhibiting the formation of large flakes^34^. The SEM images also show plucked fibre cavities, smooth worn fibre surface, filler clusters and broken fibres. These features reveal that the inclusion of fibre in the biocomposite increases the complexity of the wear mechanism. The smoothing of the fibre surface and the reduction of flake formation indicate that the applied stress from the antagonist is dominated by the fibres rather than the resin matrix^35^. Hence, the degree of resin matrix damage for Group L is lower compared to Group K. Nevertheless, the addition of fibre by 5 wt% does not reduce the specific wear rate of the overall biocomposite. The SEM images for Groups M and N display more flakes, and debris compared to Group L. Microcracks, flakes, and mild fibre-matrix debonding were observed around the fibres in Group M and N.

Spalling pits were distributed randomly on the wear track for Group N. The formation of spalling pits is related to the effects of fibre clustering, detachment of fibre clusters, and abrasive wear^36^. As the content of fibre increases, the potential for inevitable fibre clustering, microcrack initiation and fibre-matrix debonding increases. These factors reduce the effectiveness of the fibre interlocking the resin matrix. The fibre clusters tend to detach from the resin matrix, leaving pits on the wear track. Embedded fibres on the wear track contribute to the three-body abrasive wear mechanism. As the rotary wear progresses, more pits develop on the resin matrix and antagonist. The microcracks were initiated at the resin-fibre interface, which has poor bonding interactions and low fatigue strength. Fractured particles and debris were built up into shielding areas behind fibres and formed flakes. The flakes attached to the resin composite lead to adhesive wear. The qualitative SEM analysis of the wear tracks revealed that an excessive amount of short-glass fibre in Groups M and N reduces the effective cohesive forces between the resin particles and increases the tendency of fatigue fracture on the worn surface^36^.

### Wear track subsurface morphology using SEM

**Figure 9:**
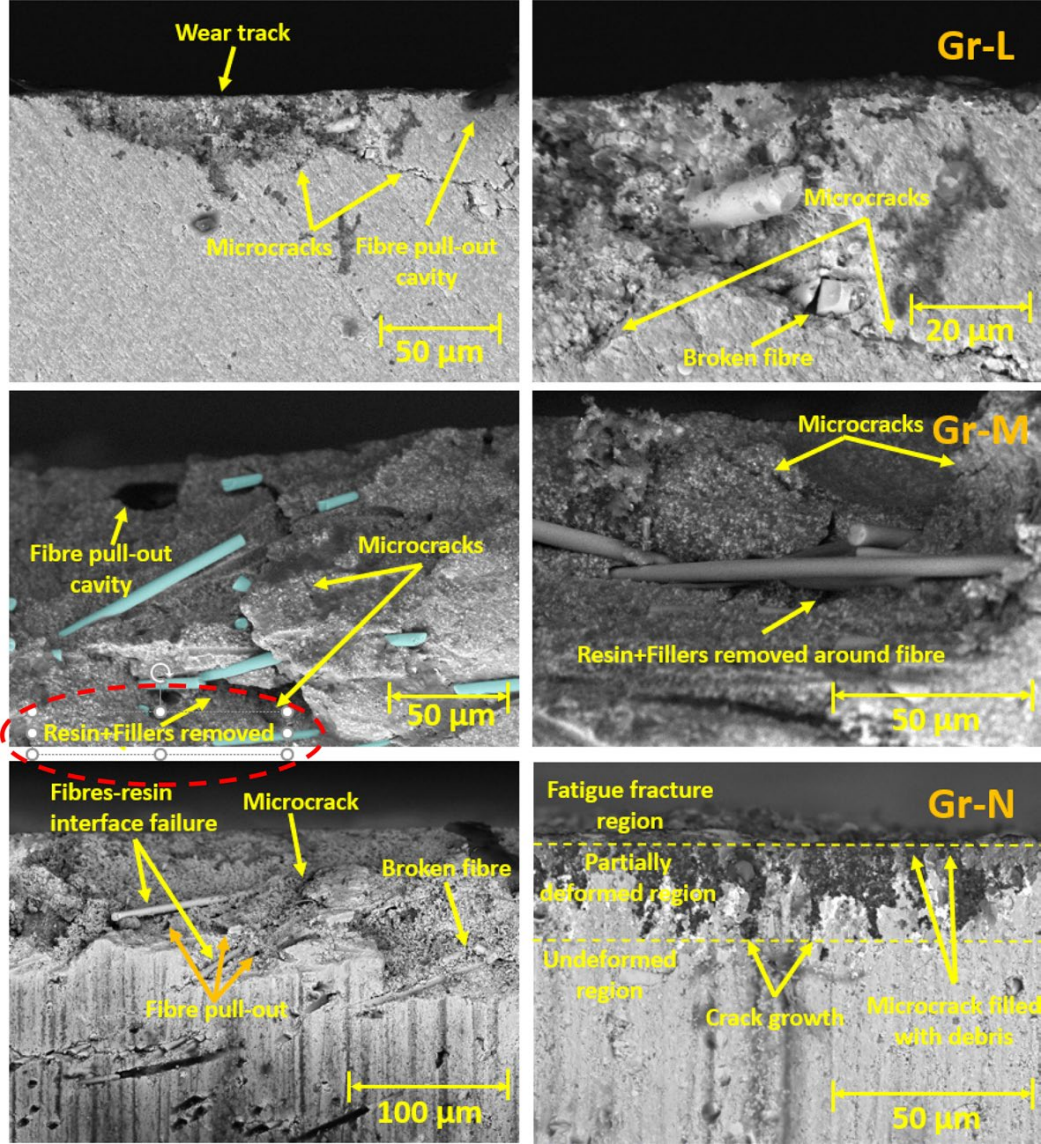
Wear track subsurface SEM images for Rotary Test: 5% 50AR, 5% 70AR and 5% 100 AR.

Figure 9 compares the SEM images of rotary wear track cross-sections for fibre-reinforcing resin composites (Group L-N). Microcracks and fibre pull-out cavities were observed in all Groups. The SEM images for Group L and M show the microcracks propagate inward and change direction around the fibres. The path length of the microcracks exceeds 50 μm. There is no clear evidence that the presence of fibre interlocks the resin matrix and fillers. However, the formation of fibre pull-out cavities and fibre-resin interface failure reveal poor bonding interactions between the resin-fibre interface. High fatigue stress on the wear surface cause broken fibres, crack growth, and removal of resin and filler particles. This phenomenon is shown in the SEM images of Group M, where a high degree of crack growth results in the removal of resin and filler particles around the fibres. The exfoliated particles may deposit on the wear track, resulting in three-body abrasive wear^36^. Therefore, Group M exhibits a higher rate of resin matrix damage compared to Group L and N, which is consistent with the rotary wear test findings. Surprisingly, the SEM images of Group N show most of the microcracks filled with debris, propagate inwards and parallelly. Compared with the microcracks observed in the SEM images of Group L and M, Group N has shorter microcrack path lengths (< 40 μm). This is attributed to the high fibre-to-filler (15 wt% fibre) content in the resin matrix limits the microcrack propagation in the vertical direction.

**Figure 10:**
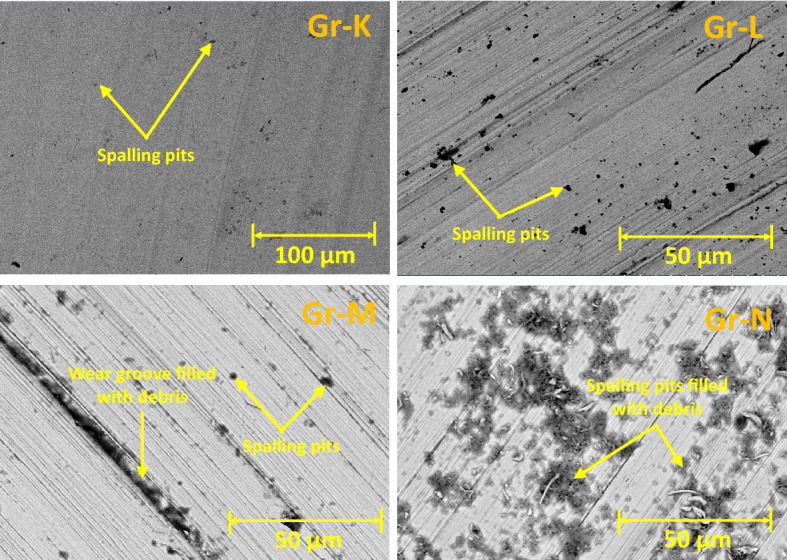
SEM images of antagonist (stainless steel ball) wear surface for Rotary Test (Groups K-N): No Fibre, 5% 50 AR, 5% 70 AR and 5% 100 AR, showing an increase in the spalling pit area with the increasing fibre wt%.

The SEM images in Fig. 10 show the worn surfaces of the antagonists caused by the rotary wearing (Group K-N). Wear grooves and spalling pits were observed in all Groups. Group K (no fibre) has smaller spalling pits compared to Group L-N (with fibre). This reveals that the presence of fibre in resin composite plays a significant role in abrasive wear compared to filler. As the content of fibre increases from 5 to 15 wt%, the size of the spalling pits increases. Debris was found within the wear groove and spalling pits. The SEMs indicate that the debris is made up of a mixture of filler and fibre fragments. This suggests that the rotary wear system involves three-body abrasive wear and adhesive wear mechanisms.

**Figure 11:**
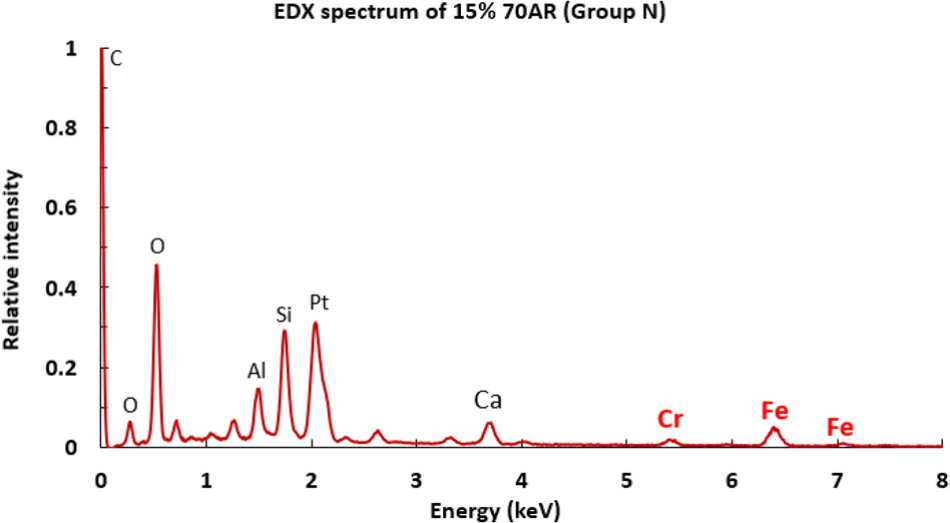
EDX analysis of Group I sample.

EDX analysis of a Group I sample is shown in Fig. 11. Peaks of Chromium and Iron (Fe) are seen, showing that the steel debris has been deposited on the biocomposite sample surface.

## Discussion

The structural responses of multifunctional materials to mechanical forces are complex because they are composed of the mechanical properties of their constituent materials. The process gets more complicated during the wear phenomenon where several mechanical, chemical, and thermal interactions occur within the multifunctional material, antagonist, and surrounding fluid^8,37^. Based on the aforementioned results, the mechanical interaction between the biocomposites and antagonist stainless-steel balls in the modified Fusayama solution bath was further discussed in this section. Methacrylate-based biocomposites are inherently brittle and abrasive wear is the dominating mode against the stainless-steel antagonist. However, once the fibres are added, the material structure and the response to the external loads are more complex. The rotary studies in this test were conducted to confirm the reciprocating test results for specific wear rate and wear phenomenon.

The variation of CoF with time and the average CoF depends on the shape of the contact area, applied normal load, lubricating conditions, and shape and size of debris particles. In the current study, the fibre-to-filler weight fraction had a significant influence on the average CoF, whereas the fibre AR seemed to have minimal impact. The average CoF increases as the fibre-to-filler weight fraction increases. The finding is consistent with the earlier studies^20^. This could be due to all three fibre lengths being smaller than the critical fibre length, which can reduce fibre-resin interfacial bond strength. For Group A, CoF rises rapidly and had a lower steady-state value with a shorter transient period. With the replacement of fillers with fibres for Groups B to E, the material structure changed, and the time taken to reach the steady state become longer. The transient period of Group-A is short wherein the CoF rises rapidly and had a lower steady-state value. Higher CoF is seen with the addition of glass fibres from 5 to 15 wt%. Also, the variation of fibre AR from 50 to 100 had minimal effect on the average CoF for the tested Groups. The rotary wear test shows a similar trend of CoF as the reciprocating wear test as the fibre-to-filler weight fraction increases. Since the rotary wear test creates a centrifugal force that pushes debris particles away from the centre of the circle wearing path, the quantity of debris particles trapped within the wear track is reduced. As a result, the effect of the three-body abrasion wear mechanism on the biomaterial is lower for the rotary wear test. Therefore, the average CoF for the rotary wear test is relatively lesser compared to the reciprocating wear test for the same fibre-to-filler weight fraction and fibre AR biocomposite. This phenomenon also affects the specific wear rate; rotary wear-tested biocomposites exhibit a lesser wear rate compared to reciprocating wear test.

Generally, under artificial saliva lubrication, the contribution of adhesive wear is minimal^28^. However, under higher contact pressure of the mating surfaces, as shown in Fig. 8, flakes are formed. It seems abrasive wear was followed by adhesive wear as the deep grooves of the wear tracks were filled with debris and pressed into the wear grooves in the form of flakes. This prevented further matrix removal through adhesive wear. Though the transfer film aspect is considered, the contact pressure is too high in the current study which exceeds the transfer film resistance. Also, Fatigue wear generally originates from stress concentrations such as voids, scratches and dissimilar material interfaces^38^. Cracks are generated either on the surface or subsurface which could grow and dislocate the local material at the microscopic/macroscopic scale in the form of debris^19^. All the tested Groups demonstrated wear grooves (ploughing) along with debris where the soft resin and reinforcements are removed by the asperities of the antagonist as seen in SEM images in Figs. 4, 5, 6, 7 and 8. Group-A had several partially detached flake-shaped wear particles (Fig. 4, arrowed) which are developed due to shearing of the softer polymer caused by the interaction of normal and tangential loads on the surface^39^ and/or subsurface crack propagation^20^. For Groups B-J, the SEM images (Figs. 5, 6, 7) revealed that the resin matrix removal during the wear process resulted in glass fibres being exposed to the antagonist, which were eventually fractured and detached from the resin matrix. Wear tracks of all tested Groups show the matrix cracking along with debris and filler clusters. Composites in Group B show matrix wear around the fibre, exposure of fibres on the surface, fibre thinning and fibre breakage (Fig. 7). Fibre debonding is evident in the wear tracks of Groups C, D, F, H, I and J. Fibre removal/plucking is seen in all Groups which creates a cavity which in turn increases CoF and higher material removal. The wear track surfaces also contain flakes (a general characteristic of fatigue wear) and debris-filled cavities (Figs. 7, 8, 9). The flakes act as concealment for the underneath resin matrix and protect the resin matrix from tangential abrasion. For Groups I-J with 15 wt% fibres, a higher number of partial flakes and compressed debris is seen which reduced the amount of wear on the surface. The SEM images show no fibre residue on any of the sample surfaces. This is due to such residue being washed away by the modified Fusayama solution during both the reciprocating and rotary wearing process.

SEM analysis of the wear surfaces revealed to be abrasive and fatigue wear, where the material on the surface is removed or displaced in the micro/nanoscale, increasing the surface roughness. For example, in the case of dentistry, this high surface roughness of the occlusal surface of a tooth restoration creates microcavities that promote bacterial colonies^3^ and forms plaque, reducing the structural integrity of the restoration^40^. The measured mean post-wear surface roughness values of the tested Groups were similar to those for amalgam^41^ and particulate-based biocomposites^11,42^. Post-wear surface roughness in the current study demonstrated an increase for 5 and 10 wt% fibre loading for all three fibre lengths and a reduction for 15 wt% loadings. The reduction for 15 wt% loadings can be attributed to the presence of compressed debris masking the wear grooves and cavities of plucked fibres which were lower for the 5 and 10 wt% Groups, but higher for the 15 wt% Groups as seen in the SEM images in Figs. 6, 7, 8 and 9. The 5 and 10 wt% Groups which had a higher quantity of glass particles in the composite are likely to prevent the deposition of debris in the wear grooves.

Based on the SEM analysis of the wear surfaces, the wear mechanism of the tested short glass fibre reinforced bio composites could be summarised as (i) removal of matrix material due to abrasion by the antagonist; (ii) weakening of the interfacial bond between the resin and reinforcement (glass particles and fibres)^43^; (iii) dislodging of filler particles; (iv) exposure of glass fibres due to the detachment of surrounding filler and matrix; and (v) fibre thinning and/or breakage (cantilever action) due to further abrasion. The finding is in agreement with the earlier studies of Zhang et al. and Ozturk and Ozturk^44,45^.

Although the qualitative SEM analysis of the wear track surface allows us to understand the effects of fibre-filler fraction and wear on the evolution of microstructure, it does not provide comprehensive information to understand the wear mechanism, the subsurface damage of the resin matrix, and correlation of the wear track microstructure with the specific wear rate. Hence, the wear cross-section of the tested biocomposite Groups was also observed via SEM and they show the appearance of fibre pull-out cavities, microcracks, and fibre-resin interface failure. These provide additional information about the path length and direction of microcrack propagation within the biocomposite matrix, fibre-resin interfacial adhesive and interlocking properties. Group N has the highest fibre-to-filler weight fraction, but it has a lower specific wear rate compared to Group M. The SEM images show that Group N has a shorter microcrack path length compared to the Group M. Severe fatigue fracture takes place on the top wear surface. Partial plastic deformation occurs under this surface. The degree of plastic deformation decreases with depth. The vertical crack elongation may involve crack initiation, crack growth from top to bottom, and crack termination by fibres and fillers. The accumulation and compression of debris in the microcrack voids cause the microcracks to expand in all directions. Since the propagation direction and the path length of the microcrack is being limited, Group N has a lower wear volume compared to Group M. The SEM findings consistent with the specific wear rate results.

Past investigators have observed a direct correlation between the specific wear rate and fibre weight fraction^20^ for glass fibre reinforced composites that did not contain particulate reinforcements. However, for the present composites which contained particulate reinforcements in the range of 40–55 wt%, the wear rate increased from 5 to 10 wt% of fibres but decreased for 15 wt% of fibres. Several researchers^8,46,47^ have proposed a “protection hypothesis” to justify a reduction in specific wear rate with an increase in fibre fraction. That is, the weak resin-rich area around the fibres (due to mismatch in the geometries) is more rapidly abraded, creating a void surrounding the fibre. The displaced 0.7 µm filler particles get embedded in the micro-pores on the wear surface as well as in the voids surrounding the glass fibres. The compressed debris increases the stiffness of the material locally and hence reduces the wear rate. Higher wt% of fibres are also known to increase crack bridging and crack deflection capabilities, thereby enhancing the fracture toughness and fatigue performance^48^. The key contributor to the low specific wear rate for Groups H-J is likely to be high fracture toughness, as reported by Heintze et al.^49^ and Kim and Watts^4^.

The difference in the material hardness between the biocomposite components and the steel ball also is an influencing parameter in this study. The hardness of stainless-steel balls is 190 HV (3 Moh)^50^ whereas the hardness of S-Glass fibre is 900 HV (6.5 Moh)^51^. Since the S-Glass fibre is harder than the steel ball, the glass fibre in the biocomposite could abrade the steel ball and the degree of abrasion increases as the fibre-to-filler weight fraction increases. This can be seen as the shallower and wider wear trajectory observed on the biocomposite (Fig. 1a,b) and the enlargement of the spalling pits on the steel ball (Fig. 10) as the fibre-to-filler weight fraction increases. Hence, the fibre-to-filler fraction in the biocomposites is the main factor affecting the abrasion and fatigue mechanism of the wear test compared to fibre AR.

## Conclusion

A systematic in-vitro study on the wear performance of biocomposites filled with various fibre-to-filler weight fractions and fibre AR was carried out in this investigation. The reduction of particulate filler fraction and an increase in the fibre fraction provides a unique tribological behaviour that helps in optimising the material composition for biocomposites. Within the limitations of the study, the average CoF of biocomposites was primarily affected by the fibre-to-filler weight fraction. The transient period for CoF was longer for 5 wt% fibres compared to 10 and 15 wt%. Steady-state CoF depended primarily on the fibre weight fraction in comparison with fibre length. Specific wear rate increased from 5 to 10 wt% but decreased to 15 wt%. 15wt% Groups were also supported by the compressed debris filling the wear grooves, reducing the surface roughness and subsequently reducing the wear rate. It was seen that fibre weight fraction had a higher influence on the specific wear rate than the fibre length. SEM observations revealed the wear mechanism initiated as matrix-filler debonding, removal of the matrix due to abrasion followed by filler particles and finally thinning and/or breakage of glass fibres. Based on this wear study, compositions with 5 wt% 50 AR and 5 wt% 70 AR were found to have lower specific wear rates while maintaining flowability compared to other Groups.

The SEM analysis of the wear track top view provides insight into the wear groove, spalling pit, worn fibre and worn resin matrix features on the antagonist contact surfaces. These allow us to compare the degree of wearing on fibre and resin matrix, and correlate to the distribution of applied stress on both surfaces. The SEM analysis of the wear track cross-section provides additional information about the wear mechanism, and subsurface damage of the resin matrix, and allows us to correlate the microcrack path lengths with the specific wear rate. Hence, complementary SEM analysis of wear track surface and cross-section are conducted to obtain comprehensive microstructural information and understand the wear behaviour of biocomposites. Clinically, the wear of biocomposites is a multifunctional parameter in the complex bio environment that cannot be explained by a single set of parameters such as fibre loading and fibre length alone. The current comprehensive in-vitro wear study correlates the mechanical and microstructural properties of biocomposites with the fibre-to-filler weight fraction and fibre AR. This would help to understand the biocomposite wear behaviour.

## Data Availability

The datasets used and/or analyzed during the current study are available from the corresponding author on reasonable request.
